# High temperature pre-digestion of corn stover biomass for improved product yields

**DOI:** 10.1186/s13068-014-0170-2

**Published:** 2014-12-03

**Authors:** Roman Brunecky, Sarah E Hobdey, Larry E Taylor, Ling Tao, Melvin P Tucker, Michael E Himmel, Stephen R Decker

**Affiliations:** Chemical Biosciences Center, National Renewable Energy Laboratory, 15013, Denver, West Parkway, Golden, CO 80401 USA; National Bioenergy Center, National Renewable Energy Laboratory, 15013, Denver, West Parkway, Golden, CO 80401 USA

**Keywords:** Biomass, Pretreatment, Enzymatic hydrolysis, CelA, E1, *Caldicellulosiruptor bescii*, *Acidothermus cellulolyticus*, *Thermotoga maritima*

## Abstract

**Introduction:**

The efficient conversion of lignocellulosic feedstocks remains a key step in the commercialization of biofuels. One of the barriers to cost-effective conversion of lignocellulosic biomass to sugars remains the enzymatic saccharification process step. Here, we describe a novel hybrid processing approach comprising enzymatic pre-digestion with newly characterized hyperthermophilic enzyme cocktails followed by conventional saccharification with commercial enzyme preparations. Dilute acid pretreated corn stover was subjected to this new procedure to test its efficacy. Thermal tolerant enzymes from *Acidothermus cellulolyticus* and *Caldicellulosiruptor bescii* were used to pre-digest pretreated biomass at elevated temperatures prior to saccharification by the commercial cellulase formulation.

**Results:**

We report that pre-digestion of biomass with these enzymes at elevated temperatures prior to addition of the commercial cellulase formulation increased conversion rates and yields when compared to commercial cellulase formulation alone under low solids conditions.

**Conclusion:**

Our results demonstrating improvements in rates and yields of conversion point the way forward for hybrid biomass conversion schemes utilizing catalytic amounts of hyperthermophilic enzymes.

## Background

A key technical barrier to commercializing any biofuel (alcohol or hydrocarbon) or chemical from biomass via a sugar platform is the high cost and relative inefficiency of producing fermentable sugars from lignocellulosic biomass [1,2].

After thermochemical pretreatment of biomass, the resulting mixture must be cooled before commercial cellulases and/or conversion microbes are introduced. The current upper limit for commercial cellulases/hemicellulases is about 50°C (sequential hydrolysis and fermentation or SHF processes) and about 38°C for microbes (simultaneous saccharification and fermentation or SSF processes) [3-5]. Active cooling requires additional energy input and/or cooling water, whereas passive cooling is relatively inexpensive, but requires time to be effective [6]. In the case of thermophilic enzymes, we can take advantage of this inherent process cooling time in conjunction with the latent heat of mixing to “jump-start” the enzyme hydrolysis process, similar to high temperature gelatinization in starch hydrolysis. Employing a higher operating temperature hold step will enable thermophilic enzymes to be added earlier in the process, resulting in both time savings and improved conversion efficiency compared to using current mesophilic, commercial enzyme cocktails.

To evaluate this proposed process, we employed a 24-h, high temperature enzymatic pre-digestion step with a variety of thermophilic enzymes, followed by a conventional digestion step with a commercial enzyme cocktail. The thermophilic cellulases utilized in this case were a combination of known, thermal tolerant cellulases from *Acidothermus cellulolyticus* (E1 endoglucanase, T^o^_opt_ about 80°C) and *Caldicellulosirupter bescii* (CelA, T^o^_opt_ about 85°C) [7-10]. Additional enzymes tested included a partially purified *C. bescii* culture broth containing predominantly CelA; as well as *C. bescii* xylanase enzymes and their fragments supplemented with a thermal tolerant β-D-glucosidase from *Thermotoga maritima* [11,12]*.*

## Results and discussion

### Low solids saccharification

We initially used a high temperature enzyme loading of about 15% of the total enzyme loading to test the efficacy of the high temperature hold step at a low solids loading. The digestion conditions were 50 mM sodium acetate buffer, pH 5.5 with 100 mM NaCl, 15 mM CaCl_2_ with the enzyme loadings listed below in each figure loaded on a milligrams per gram of glucan basis.

**Figure 1:**
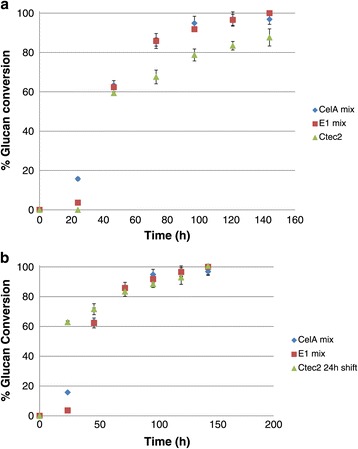
**Comparison of biomass conversion with and without a high temperature hold step. a**: Initial high temperature hold experiment. CelA mix: 3 mg CelA/g glucan, 1 mg E1/g glucan, 0.5 mg β-D-glucosidase/g glucan, and 16 mg CTec2/g glucan. E1 mix: 3 mg E1/g glucan and 17 mg CTec2/g glucan. CTec2: 20 mg CTec2/g glucan. **b**: Initial high temperature hold experiment with CTec2 shifted 24 h. CelA mix: 3 mg CelA/g glucan, 1 mg E1/g glucan, 0.5 mg β-D-glucosidase/g glucan, and 16 mg CTec2/g glucan. E1 mix: 3 mg E1/g glucan and 17 mg CTec2/g glucan. CTec2: 20 mg CTec2/g glucan.

The high temperature hold digestion yielded approximately 10 to 15% higher glucan conversion at most time points observed (Figure 1). These results were subject to a one tailed homoscedastic T-test, and found to be significant (p <0.05). We also see that compared to the CTec2-only digestion, the high temp hold samples reach higher extents of conversion at earlier time points, this phenomenon is observed even if the CTec2 curve is shifted 24 h earlier (i.e., same start time), although the improvements in conversion are smaller (Figure 1b).

### Enzyme loading optimization

**Figure 2:**
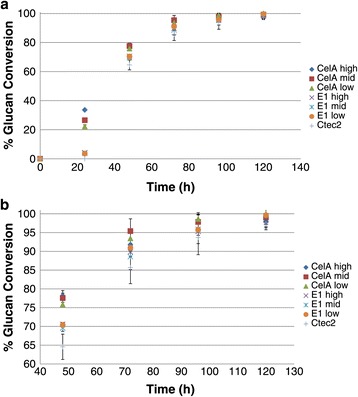
**Opimization of the high temp hold step. a**: High temperature hold optimization. The digestion conditions are 50 mM acetate buffer, pH 5.5 with 100 mM NaCl, 15 mM CaCl_2_ with enzyme loadings listed below. * Denotes a statistically (*P* < .05) relevant improvement in conversion compared to CTec2 at 120 h. High CelA mix: 5 mg *C. bescii* broth/g glucan, 1 mg E1/g glucan, 0.5 mg β-glucosidase/g glucan, and 13.5 mg CTec2/g glucan. Mid CelA mix: 3 mg *C. bescii* broth/g glucan, 1 mg E1/g glucan, 0.5 mg β-glucosidase/g glucan, and 15.5 mg CTec2/g glucan. Low CelA mix:* 2 mg *C. bescii* broth/g glucan, 1 mg E1/g glucan, 0.5 mg β-glucosidase/g glucan, and 16.5 mg CTec2/g glucan. Low E1 mix: 3 mg E1/g glucan, 0.5 mg β-glucosidase/g glucan, and 16.5 mg CTec2/g glucan. Mid E1 mix:* 4 mg E1/g glucan, 0.5 mg β-glucosidase/g glucan, and 15.5 mg CTec2/g glucan. High E1 mix: 6 mg E1/g glucan, 0.5 mg β-glucosidase/g glucan, and 13.5 mg CTec2/g glucan. CTec2: 20 mg CTec2/g glucan. **b**: High temperature hold optimization close-up of 48 h + digestion points to better illustrate differences.

**Figure 3:**
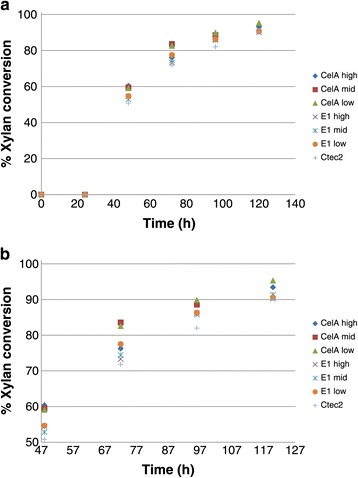
**Xylan conversion.** The digestion conditions as described in Figure 2 **a**. We note here that while all CelA loadings are statistically significantly better than the control at the 96-h mark, only the CelA high loading showed a statistically (*P* < .05) relevant improvement in conversion compared to CTec2 at 120 h. **b**: High temperature hold optimization close-up of 48 h + xylan digestion points to better illustrate differences.

To optimize the thermophilic enzyme loadings, we assayed high, mid-range, and low thermophilic enzyme loadings for the high temperature hold, while maintaining the total enzyme loading at 20 mg/g glucan. Optimization of conditions for the high temperature hold indicate that higher loadings of the thermophilic enzymes containing CelA provide stronger initial boosts to the glucan conversion (24 h) (Figure 2a) and also provide subsequent 4 to 7% enhancements in conversion when comparing the best CelA digestions to CTec2 digestions (Figures 2a and 2b). These results were subject to a one-tailed homoscedastic *t*-test, and found to be significant (*P* <0.05) for the CelA low and E1 mid loadings at 120 h. This improvement is still observable for the other high temperature hold enzymes, but the extents of conversion are slightly lower and not statistically significant at 120 h. This result may be due, in part, to the plateau effect that occurs toward the latter extent of conversion time points; however, if one examines the data on a time-to-target basis, the high temperature hold provides clear benefits, achieving equivalent extents of conversion 24 h or more prior to the CTec-2 control conversion curve. This 20% reduction in processing time will improve the economics of large-scale biomass conversion processes. Also noteworthy is the determination that xylan conversion displays a similar, although slightly less dramatic, effect in conversion extents, achieving a 10% improvement at early time points and approximately 3 to 4% at the digestion endpoint. These data are statistically significant (*P* <0.05) for all CelA loadings at 96 h, and significant only for the CelA high loading at 120 h (Figure 3).

### High solids stepwise saccharification at higher enzyme loading

**Figure 4:**
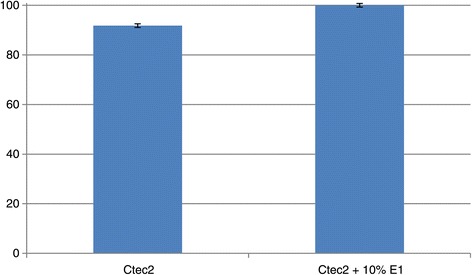
**20% High solids validation test.** CTec2 loaded at 40 mg/g biomass. CTec2 + E1 loaded at 4 mg/g biomass E1 and 36 mg/g CTec2.

To validate our low solids loading results, we ran the high temperature hold experiments on a limited scale using 20% solids loading conditions. The total enzyme loadings were 40 mg CTec2/g glucan or 36 mg CTec2/g glucan and 4 mg E1/g glucan. The results are reported in Figure 4. Our results show that a stepwise digestion with E1 increases the glucan-to-glucose conversion by approximately 10% on washed solids pretreated corn stover (PCS) compared to samples that were pre-incubated without E1 (Figure 4). This is especially significant, considering that samples containing E1 permitted a 10% lower CTec2 loading. These results are consistent with those results obtained in the low solids loading cases.

### Techno-economic analysis

Minimum ethanol selling price (MESP) values were estimated using our results from the initial high temperature hold experiment (Figure 2). These MESP results are summarized in Table 1. The MESP decreased steadily as the enzymatic hydrolysis time was increased; a result we feel is mainly due to an increasing yield of monomer glucose. Experimental data were taken every day to verify enzymatic hydrolysis yield, or monomer glucose yield. Initially, the CelA mix showed a lower initial yield compared to CTec2 (4 d) alone; however, starting at day 4, we see an improvement in the yield results. We have included a hypothetical comparison with CTec2 showing the effect of starting the digestion at the same time as the pre-digestion with CTec2 (5 d), which assumes an instant cooling scenario, where the CTec2 enzyme can be added immediately. We can see from these studies that there is a benefit of 14 to 18 cents per gallon of ethanol produced from utilizing the high temperature hold step for the best case of the CelA-containing mix compared to the CTec2 digestions, depending upon whether or not there is a 24-h delay in starting the CTec2 digestion as the biomass cools.

**Table 1:** **Minimum ethanol selling price**

**MESP ($/gal)**			
	**3 day**	**4 day**	**5 day**
CelA mix	$2.50	$2.22	$2.12
E1 mix	$2.52	$2.21	$2.15
CTec2 5 d	$2.44	$2.30	$2.26
CTec2 4 d	nd	$2.44	$2.30

## Conclusions

A high temperature hold step utilizing thermophilic or hyperthermophilic enzymes shows promise in a biomass conversion process where gradual cooling (dilution/neutralization cooling only or air cooling) of biomass is utilized. We observe approximately 10 to 15% improvements in conversion at given time points compared to CTec2 digestion alone in these specific cases. In a process scenario where biomass cooling is rapid, such as when active cooling is utilized, the gains are less impressive, perhaps a 5% improvement at best. However, endpoint conversions are still typically a few percent higher compared to the mesophilic CTec2 cocktail alone. This may be in part due to possible synergistic mechanisms between the novel mechanism of the CelA-based cocktail, which creates cavities within the biomass, and the more conventional surface ablation hydrolysis model displayed by fungal enzymes [7]. However, the E1-only cocktail demonstrates almost an identical improvement, and the mechanism of E1, an endoglucanase, is likely to be primarily increasing the number of reducing ends available for the cellobiohydrolases in subsequent fungal cocktail digestion. We suggest that these enzymes exhibit different modes of synergy with the commercial fungal enzyme cocktail. Our proposed high temperature hold process may be in some ways compared to the industrial application of thermophilic α-amylases utilized in the modern dry grind process for corn starch-derived ethanol. In that process, addition of hyperthermophilic α-amylases (Takatherm®) to the corn mash immediately following cooking in the hydroheaters has dramatically improved conversion rates for soluble sugar production. However, given the major differences between the two feedstocks, especially that of the insolubility of lignocellulose, we expect less dramatic improvements for our process compared to starch liquefaction and conversion.

We have determined that low loadings of the high temperature cellulose cocktails can provide better improvements in both rates of digestion and final glucan conversion extents compared to CTec2 alone. The proposed improvements from this study have been validated for both low solids conditions and at endpoint conversion conditions for high solids. Techno-economic analysis using our results suggests that a significant savings is achievable due to the reduced processing times and improved extents of conversion. If the results can be validated on a large scale, they should provide a roadmap to improved conversion yields and reduced processing times for current biomass saccharification schemes, and will be drop-in compatible with existing plant configurations.

## Materials and methods

### Biomass for high and low solids digestions

Pretreated biomass was obtained from the 2012 state of technology production run in the NREL Integrated BioRefinery Facility (IBRF) pilot plant. Briefly, Pioneer 33B51 tub ground corn stover was received in 2003 from Wray, CO (Kramer Farm) and was further knife milled (Jordan Reduction Systems) through a ¾-inch rejection screen in the NREL IBRF. Corn stover deacetylation and sulfuric acid impregnation was performed in the 1900-L Dynamic Impregnator (DI) tank (American Process Systems, Gurnee, IL). Dry corn stover was added to the tank along with a dilute sodium hydroxide solution (0.4 w/w) to deacetylate the material prior to pretreatment. The material was dewatered, neutralized, acid impregnated, and pretreated. The pretreatment conditions utilized were 160°C, 0.8% (w/w) H_2_SO_4_, and 10 min in a large horizontal pretreatment reactor (Mesto Inc., Norcross, GA) configured for two-tube operation (Pretreatment ID P120927) [13]. The biomass was washed with water by centrifugation until a pH of 5 was observed. The biomass composition was analyzed as previously described and is listed in Table 2 [14].

**Table 2:** **Composition analysis of process relevant biomass**

**Sample ID**	**% Ash**	**% Protein**	**% Lignin**	**Lignin corrected?**	**% Glucan**	**% Xylan**	**% Galactan**	**% Arabinan**	**% Fructan**	**% Uronic acid**	**% Acetate**	**Total %**
P120927 DCS (high and low solids)	4.07	ND	23.9	No	63.9	5.0	0.6	0.8	0.00	ND	0.2	98.5
XT110613 A (high solids)	5.12	ND	26.8	No	60.2	2.9	0	0.9	0.2	ND	0.4	96.6

### Biomass for high solids digestions

Corn stover biomass was prepared and pretreated as follows: Pioneer maize variety 33A14 whole stover from the Kramer Farm in Wray, CO was pretreated in the 200-kg/day continuous, high-solids, pilot-scale horizontal pretreatment reactor system at NREL using 2% sulfuric acid at 158°C with a residence time of 5 min (Pretreatment ID XT110613). Deacetylation of the stover was performed using 0.1M NaOH at an 8% solids loading at 80°C, and mixed at 15 rpm for 2 h prior to dilute-acid pretreatment. The “washed PCS” was washed with water by centrifugation until a pH of 5 was observed. The washed PCS was air-dried on the bench top with manual mixing in order to achieve up to 20% solids in the final reaction vessel (after the addition of enzyme solutions and buffer). The biomass was analyzed as previously described [14].

### Enzymes

The enzyme mixtures utilized for the high temperature hold were selected for their abilities to operate at high temperature regimes, and the hyperthermophilic enzymes and mixtures were selected for their ability to operate at 80°C. For the hyperthermophilic conditions, we utilized mixtures of the following enzymes: the multifunctional (endocellulase, exocellulase, xylanase) CelA was from *C. bescii* [7]. CelA was supplemented with the endocellulase E1 from *A. cellulolyticus* [8], and the β-D-glucosidase from *T. maritima* was obtained from Megazyme International Ireland (Bray, Ireland)*.* We also utilized a CelA-enriched fraction of culture broth from cellulose-grown *C. bescii*, which is composed primarily of CelA, for some experiments. These enzyme mixtures were then supplemented with twice desalted Cellic® CTec2 from Novozymes (Bagsvaerd, Denmark) after the initial 24-h high temperature hold step. Details of each mixture are included in each figure.

### Time course saccharification - low solids

Stepwise saccharifications at about 2% solids loading were performed in a 1.5-mL volume in two steps: 1) high temperature (80°C) digestion using a mixture of thermophilic enzymes and 2) a lower temperature digestion (50°C) using the enzyme cocktail Cellic® CTec2. All enzyme loadings were loaded on a mass basis, that is, milligrams of enzyme per gram of biomass, with a final total loading of 20 mg/g. Samples were taken generally at 24-h intervals, and the enzymes were inactivated by boiling for 15 min. Samples were filtered through 0.45 μm Acrodisc syringe filters and analyzed for glucose, and cellobiose by HPLC. Samples of 20 μL were injected onto an Agilent 1100 HPLC system equipped with a BioRad Aminex HPX-87H 300 mm × 7.8 mm column heated to 55°C. A constant flow of 0.6 mL/min was used with 0.1M H_2_SO_4_ in water as the mobile phase to give optimal sugar separation. Glucose, xylose, and cellobiose were quantified against independent standard curves. All experiments were performed in triplicate, and the resulting extents of conversion are shown as a percentage of maximum theoretical glucan converted. Measurements were standardized to a maximum conversion of 100%, and thus, the results between Figures 1 and 2 should not be compared directly. We have also performed a one-tailed homoscedastic *t*-test on all data and reported the results as relevant in the paper.

### High solids endpoint saccharification

The initial high solids experiment was an endpoint saccharification performed in two steps: 1) high temperature digestion using purified endocellulase E1 from *A. cellulolyticus* followed by 2) a lower temperature digestion using the enzyme cocktail, Cellic® CTec2. Two-step saccharifications were performed on washed PCS (XT110613-Kramer 33A14, Table 2).

Digestions were carried out in 20-mL glass scintillation vials loaded to an equal final weight of 3.2 g biomass to maintain similar mixing characteristics. Biomass was dispensed by hand and weighed to the nearest 0.001 g. Digestions of PCS were carried out on 20% solids in a two-step format. In step 1, four mg/g glucan of purified E1 was added to the samples and incubated with shaking (100 rpm) at 70°C for 24 h. After 24 h, the temperature was lowered to 45°C for 2 h to allow the sample temperature to equilibrate. In step 2, following equilibration at 45°C, CTec2 was added to a final enzyme concentration of 40 mg/g glucan and incubated for 96 h, with shaking (100 rpm) at 40°C. Endpoint samples were taken at 120 h and frozen at -20°C to stop the hydrolysis until all incubations were complete. The samples were then boiled for 10 min to denature the enzymes and diluted to 12.0 mL with Nanopure H_2_O and filtered through 0.45 μm nylon Acrodiscs. Carbohydrate quantification was performed for five monomeric sugars (glucose, xylose, galactose, arabinose, and fructose), cellobiose, and “total oligomeric carbohydrates” using an Agilent 1100 HPLC system with a Shodex sugar SP0810 analytical column with refractive index detection. The calibration curves had eight points with a range of 0.04 to 35 g/L with a validation sample at 1.5 g/L. All components were present in the calibration standards at equal levels except for the “total oligomeric carbohydrates,” which were estimated by applying the response factor for cellobiose to all peak areas that eluted prior to glucose and includes cellobiose.

### Techno-economic analysis

Process economics analysis includes a conceptual level of process design to develop a detailed process flow diagram (based on research data), rigorous materials and energy balance calculations (via commercial simulation tools, Aspen Plus), capital and operational cost estimation (capital expenditures, CAPEX, and operating expenses, OPEX, via an in-house model using spreadsheets), a discounted cash flow economic model, and the calculation of a minimum ethanol selling price (MESP). Rigorous material and energy balance calculations are performed to quantify unit-level cost estimates. For a given set of conversion parameters, material and energy balance and flow rate information are generated using Aspen Plus process simulation software (Aspen Plus. Release 7.2, Aspen Technology Inc., Cambridge MA), assuming a defined feed rate to the biorefinery of 2,205 dry US tons of corn stover per day (2,000 metric tonnes per day). These data are used to size and cost process equipment to calculate capital expense (CAPEX) and to calculate raw material and other operating costs (OPEX).

The most recent NREL design report model has been used as the baseline for modeling the variation of enzymatic hydrolysis yields in this study [13]. The integrated process model starts from dilute acid pretreatment at a moderate to high temperature (150 to 190°C) for a short time followed by enzymatic hydrolysis and co-fermentation with recombinant *Zymomonas mobilis*. Cellulase enzyme made onsite is added to the hydrolysate at an optimized temperature for enzyme activity. The monomeric xylose from the NREL FY2012 pilot demonstration reaches over 50 g/L and monomeric glucose reaches 100 g/L after enzymatic hydrolysis [13]. If saccharification and fermentation steps are conducted at different temperatures, a cooling step is required to ensure growth of fermenting organism *Zymomonas mobilis* at anaerobic conditions. Five days are required to convert most of the cellulose and xylose to the beer liquor with >70 g/L of ethanol which is then sent to recovery and purification, which uses standard adsorption technology. The solids after fermentation are separated and combusted in a fluidized bed combustor to produce high pressure steam for electricity credits and process heat. Detailed techno-economic analysis development can be found in NREL FY2012 state of technology report as well as previous NREL design reports [15,16]. In this work, process conditions and yields are updated only to the enzymatic hydrolysis area while maintaining process modeling of the other areas, to incorporate the higher temperature enzymatic pre-digestion step with hyperthermophilic enzymes coupled to a subsequent conventional saccharification. The MESPs are then calculated based on the experimental data.

## Abbreviations

CAPEX: capital expenditures
IBRF: Integrated BioRefinery Facility
MESP: minimum ethanol selling price
OPEX: operating expenses
PCS: pretreated corn stover
SHF: sequential hydrolysis and fermentation
SSF: simultaneous saccharification and fermentation
